# A Genome-Wide Analysis of Populations from European Russia Reveals a New Pole of Genetic Diversity in Northern Europe

**DOI:** 10.1371/journal.pone.0058552

**Published:** 2013-03-07

**Authors:** Andrey V. Khrunin, Denis V. Khokhrin, Irina N. Filippova, Tõnu Esko, Mari Nelis, Natalia A. Bebyakova, Natalia L. Bolotova, Janis Klovins, Liene Nikitina-Zake, Karola Rehnström, Samuli Ripatti, Stefan Schreiber, Andre Franke, Milan Macek, Veronika Krulišová, Jan Lubinski, Andres Metspalu, Svetlana A. Limborska

**Affiliations:** 1 Department of Molecular Bases of Human Genetics, Institute of Molecular Genetics, Russian Academy of Sciences, Moscow, Russia; 2 Estonian Genome Center, University of Tartu, Tartu, Estonia; 3 Department of Biotechnology, Institute of Molecular and Cell Biology, University of Tartu, Tartu, Estonia; 4 Estonian Biocentre, Tartu, Estonia; 5 Department of Genetic Medicine and Development, University of Geneva Medical School, Geneva, Switzerland; 6 Deparment of Medical Biology and Genetics, Northern State Medical University, Archangelsk, Russia; 7 Deparment of Ecology and Zoology, Vologda State Pedagogical University, Vologda, Russia; 8 Latvian Biomedical Research and Study Centre, Riga, Latvia; 9 Institute for Molecular Medicine Finland, University of Helsinki, Helsinki, Finland; 10 Wellcome Trust Sanger Institiute, Hinxton, United Kingdom; 11 Department of Internal Medicine I, Popgen Biobank, Christian-Albrechts-University, Kiel, Germany; 12 Institute of Clinical Molecular Biology, Christian-Albrechts University, Kiel, Germany; 13 Department of Biology and Medical Genetics, University Hospital Motol and Second School of Medicine, Charles University Prague, Prague, Czech Republic; 14 Pomeranian Medical University, Szczecin, Poland; Univerity of Puerto Rico at Mayaguez, United States of America

## Abstract

Several studies examined the fine-scale structure of human genetic variation in Europe. However, the European sets analyzed represent mainly northern, western, central, and southern Europe. Here, we report an analysis of approximately 166,000 single nucleotide polymorphisms in populations from eastern (northeastern) Europe: four Russian populations from European Russia, and three populations from the northernmost Finno-Ugric ethnicities (Veps and two contrast groups of Komi people). These were compared with several reference European samples, including Finns, Estonians, Latvians, Poles, Czechs, Germans, and Italians. The results obtained demonstrated genetic heterogeneity of populations living in the region studied. Russians from the central part of European Russia (Tver, Murom, and Kursk) exhibited similarities with populations from central–eastern Europe, and were distant from Russian sample from the northern Russia (Mezen district, Archangelsk region). Komi samples, especially Izhemski Komi, were significantly different from all other populations studied. These can be considered as a second pole of genetic diversity in northern Europe (in addition to the pole, occupied by Finns), as they had a distinct ancestry component. Russians from Mezen and the Finnic-speaking Veps were positioned between the two poles, but differed from each other in the proportions of Komi and Finnic ancestries. In general, our data provides a more complete genetic map of Europe accounting for the diversity in its most eastern (northeastern) populations.

## Introduction

Identifying and understanding patterns of genetic variation within and between populations has long been the major focus of studies in human population genetics. Over the last decade, our ability to investigate population structure has been significantly enhanced by the advances in high-throughput genotyping technologies, as these allow simultaneous genotyping of hundreds of thousands of polymorphic markers. Compared with the previous methodology used in human population genetics, they enabled a new level of accuracy and power without the constraint of having to use only a few loci as a proxy for the entire genome [1], [2].

To date, there is a number of studies in which the fine-scale structure of human genetic variation have been examined at a global, continental, geographic region, single country, or even a subpopulation level [3]–[11]. European ancestry is the best studied of these aspects, for which the strongest genetic differentiation has been found between the north and south of the continent. The identified European population substructure correlated well with geography [4]–[6], [12]. Although these studies included many population samples, they mainly represented northern, western, central, and southern Europe, while populations from Eastern Europe, particularly from the European part of Russia, were less represented. The region is inhabited by ethnic Russians as well as different indigenous Finno-Ugric groups. In this study, we report an analysis of 165872 single nucleotide polymorphisms (SNPs) in four Russian populations from European Russia, as well as in populations from two of the northernmost Finno-Ugric ethnic groups: Veps and Komi.

Russians are the largest ethnic group among the European populations: more than 80 million individuals live in an area that covers more than a third of continental Europe [13]. A recent study of genetic diversity in Europe performed by Nelis et al. [5] resulted in a genetic map of the continent that had a triangular structure and showed that Russians were forming one of its vertexes, together with Polish and Baltic samples. However, the Russian population included in that study originated from a single region of the European part of Russia (Tver), even though, in the context of existing genetic data (i.e., Y-chromosome and several autosomal polymorphisms) [14]–[16], European Russians could be subdivided into at least two groups: central–southern and northern Russians.

**Figure 1:**
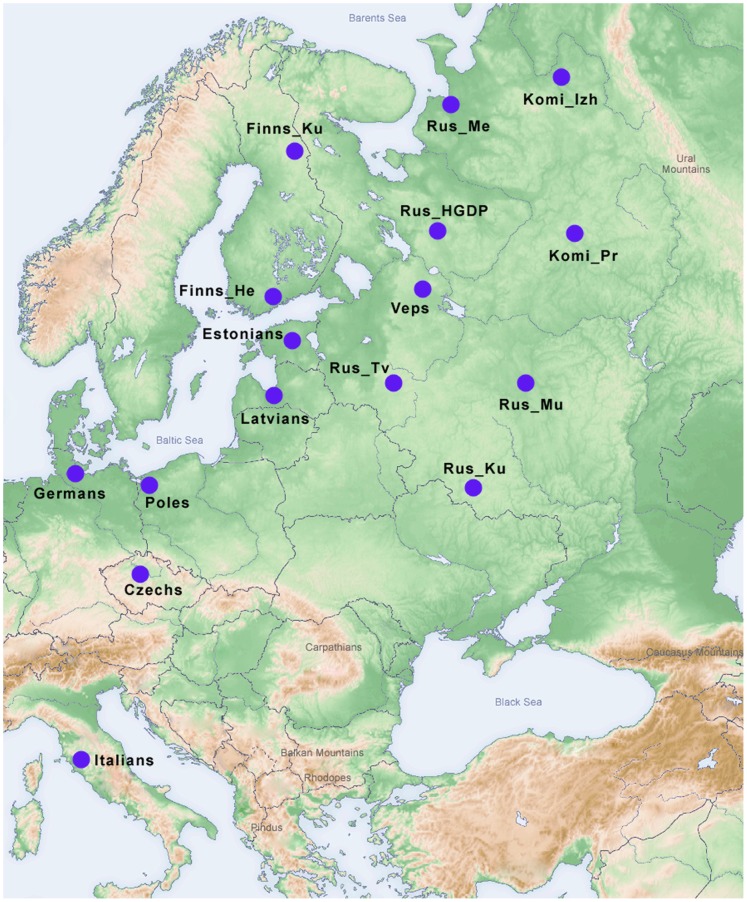
Geographic locations of the populations analyzed. Key: Komi_Izh – Izhemski Komi, Komi_Pr – Priluzski Komi, Rus_Tv – Russians from Tver, Rus_Ku – Russians from Kursk, Rus_Mu – Russians from Murom, Rus_Me – Russians from Mezen, Finns_He – Finns from Helsinki, Finns_Ku – Finns from Kuusamo, Rus_HGDP – Russians from the Human Genome Diversity Panel.

In order to study genetic structure of the European Russians in greater detail, we combined genome-wide SNP data from the Tver sample mentioned above with the genotypes of three new Russian samples from southern (Kursk), eastern (Murom), and northern (Mezen) regions of European Russia (Figure 1). Taking into account the well-documented impact of Finno-Ugric communities on the ethnogenesis of Russians [17], the genotypes of Veps and Komi were also included in our analysis. An additional reason of involving of Veps and Komi was the scarcity of the data on fine-scale genetic structure of Finno-Ugrians, which were mainly presented by Finns, Saami, Estonians and Hungarians [2], [5], [11]. The Finnic-speaking Veps (also called Vepsians or Ves in ancient times) are one of the oldest people of northern Europe that are still found in the northwest Russia (Figure 1). Veps were first mentioned in historical chronicles in the middle of the 6^th^ century [18]. It has been proposed that Veps tribes inhabited the territories between Lakes Onega, Ladoga, and Beloe as early as the first half of the first millennium [18]. In contrast to the scarce Veps, the Komi (Komi-Zyryan) people, belonging to the different linguistic branch of the Finno-Ugric family, the Permian branch, is more numerous [13], [19]. They occupy the northeastern-most location of Europe and consist of several ethnographic groups, formed during the 8^th^–19^th^ centuries [19]. We included samples from two of the geographically and socioeconomically distant Komi groups: the Izhemski Komi and Priluzski Komi [20]. Finally, to place genetic variation into the geographical context of the continental Europe, we also included genotypic data from several reference populations (Figure 1). The obtained results demonstrated similarity between Russian populations from the central part of European Russia as well as their proximity to the populations from central–eastern Europe. They were also showed that genetic peculiarity of Russians from northern Russia was resulted from their admixture with Finno-Ugric populations among them a special impact should be attributed to Komi people. It was manifested by a distinct ancestry component differed Komi from all other European populations studied.

## Materials and Methods

### Samples

The used research protocols and forms of informed consent have been approved by the Ethic Commission of the Medico-Genetic Scientific Centre of the Russian Academy of Medical Sciences (an approval was signed by the Head of the Ethic Commission, Prof. L.F. Kurilo). Written informed consent for participation was obtained from all subjects included in the study.

Blood samples were collected in EDTA-coated vacutainers after recording genealogical information and obtaining informed consent from each individual. Inclusion in the study required that all individuals belong to the native ethnic group of the region studied (i.e., they belonged to at least the third generation living in a particular geographic region), were healthy and unrelated. DNA was isolated from peripheral leukocytes according to standard techniques using proteinase K treatment and phenol–chloroform extraction [21]. Among the 615 individuals genotyped, 384 were Russians from Archangelsk (Mezen district, n = 96), Vladimir (Murom district, n = 96), Kursk (Kursk and Oktyabrsky districts, n = 96), and Tver (Andreapol district, n = 96) regions; 81 were Veps from the Babaevo district of Vologodsky region and 150 were Komi from the Izhemski (Izhemski Komi, n = 79) and Priluzski (Priluzski Komi, n = 71) districts of the Komi Republic.

DNA samples were genotyped using different versions of Illumina BeadChips: Human370CNV-Duo (Tver and Murom), Human660W-Quad (Kursk), and HumanOmniExpress (Mezen, Veps, and Komi), according to the manufacturer’s protocol (Illumina Inc., USA). All samples were subjected to the same quality control procedures using SNP and Variation Suite v.7.4.0 software package (Golden Helix, Bozeman, MT, USA). Only SNPs from the 22 autosomal chromosomes with minor allele frequency >1%, at Hardy–Weinberg equilibrium *P*>0.00001, and with genotyping success rate >95% were accepted. Cryptic relatedness was tested with the same software and from the detected relative pairs (PI >0.2), only one was chosen for the subsequent analyses at random. These steps resulted in the retention of 165,872 autosomal SNPs in 603 individuals. To investigate population genetic structure, we also included genotypes of several populations described by Nelis et al. [5]: Finns (samples from Helsinki (n = 100) and Kuusamo (n = 84), Estonians (n = 100), Latvians (n = 95), Poles (n = 48), Czechs (n = 94), and Germans (n = 100). In addition, we used free genotype data from the HapMap 3 project (Italians from Tuscany (n = 88) and Han Chinese from Beijing (n = 78) [22], and as well as from the human genome diversity panel (HGDP, Russians (n = 25) [23]. After filtering and removing all non-overlapping SNPs, a subset of 128,844 autosomal SNPs included genotypes available for all populations (except Chinese). Because background linkage disequilibrium (LD) can induce biases in principal component (PCA) [24] and structure analyses [25], both marker sets –165,872 and 128,844 SNPs – were further thinned by excluding SNPs with strong LD (pairwise genotypic correlation *r* ^2^>0.2) using a window of 200 SNPs (sliding the window by 25 SNPs at a time), which yielded 59,318 and 52,808 SNPs, respectively.

### Statistical Analysis

In order to explore the genetic structure of the populations from European Russia, several forms of analyses were performed. We started with principal component analysis (PCA), a widely used method for identifying and visualizing patterns of population structure [26]. It was carried out using the Genotypic Principal Components Analysis module of SNP and Variation Suite v.7.4.0. To obtain non-overestimated eigenvectors [27], we first ran the software using an outlier removal procedure, in which individuals with values that were greater than six standard deviations from the mean along any of the top 10 eigenvectors (principal components) were identified and removed.

Genetic differentiation among the populations was quantified by estimating pairwise Wright’s fixation indices (F_ST_) using the SMARTPCA program in the EIGENSOFT software package (v.4.2). Allele frequency differences in pairs of populations were evaluated using trend tests. The resulting *P* values were subjected to Bonferroni correction and the significance threshold was set at *P* = 0.05 (Bonferroni-adjusted *P* was equal 3×10^–7^).

Next, the population structure was examined using the ADMIXTURE software package (v.1.22), which, in contrast to PCA, implements a model-based clustering algorithm for estimating individual ancestry proportions [25]. This approach assumes that the genome of each subject originates from K unknown ancestral populations and estimates the proportions of the genome derived from each of these populations. To identify putative ancestral clusters within the samples, we ran the software assuming 2–12 subpopulations on separate runs, using default parameters. Each run was repeated at least three times to assess the stability of the clustering patterns. To validate the results, a cross-validation procedure was used [28].

Finally, to assess the potential effect of population demographics on the population structure, the runs of homozygosity (ROH) and the extent of pairwise linkage disequilibrium (LD) were examined in the populations studied. ROH in the individuals were identified using SNP and Variation Suite v.7.4.0. ROH was defined as a sequence of at least 25 consecutive homozygous SNPs spanning at least 1500 kb, with a maximum gap of 100 kb between adjacent SNPs and a minimum density of 1 SNP per 50 kb [29]. Taking into account the limited number of SNPs tested, we also used another definition of ROH, in which the limitations on the maximum distance between SNPs and the minimum density of SNPs were excluded [30], [31]. For comparative purposes the results obtained were summarized by the calculation of means for the number of ROH and the cumulative length of ROH per individual for each population. The extent of pairwise linkage disequilibrium (LD) was calculated as the genotype correlation (r^2^) between marker pairs located less than 100 kb apart using the PLINK v. 1.07.29 software [32]. A custom Perl script was applied to categorize the r^2^ values according to intermarker distances (0–5 kb, 5–10 kb, etc.) and a mean r^2^ was calculated for each category.

## Results

**Figure 2:**
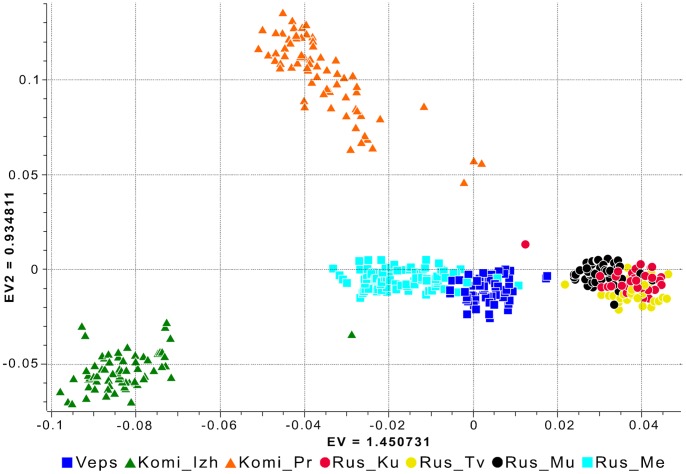
Principal component analysis of the autosomal genotypic data of individuals from European Russia. The first two PCs are shown. Each individual is represented by a sign and the color label corresponding to their self-identified population origin. Population designations are the same as in Figure 1.

To probe population structure, we first analyzed our data sets using a model-free ancestry PCA. In Figure 2 we plotted the first two principal components (PC) that had the highest eigenvalues (Figure S1). The plot demonstrated the presence of significant differences between Russian populations from the central part of the Russian Plain (i.e., populations from the Kursk, Murom, and Tver regions), which formed a single cluster on the PC plot, and the Russian population from the northern Archangelsk region (Mezen Russians). Mezen Russians exhibited closer relationships with the population of Veps. The samples of Izhemski and Priluzski Komi were located distantly, not only from Veps and Russians, but also from each other.

The lack of separation between populations from the Kursk, Murom, and Tver regions in the PC plot was consistent with the results of the assessment of population differentiation via the calculation of pairwise F_ST_ statistics, in which F_ST_ values were not greater than 0.001 (Table 1). The pairwise F_ST_ value between these populations and Mezen Russians was 0.006. The same F_ST_ value characterized the genetic relationships between Mezen Russians and Veps. This finding correlated with the population substructure observed in a plot of PC3 versus PC4, in which Mezen Russians and Veps were clearly separated from each other along PC4 (Figure S2). The highest pairwise F_ST_ estimates were obtained from comparisons that included Komi samples.

**Table 1:** F_ST_ values and the number of SNPs with significant differences in allele frequencies between the populations from Russia*.

	Rus_Tv	Rus_Ku	Rus_Mu	Rus_Me	Veps	Komi_Pr	Komi_Izh
Rus_Tv	–	0	0	172	64	262	683
Rus_Ku	0.000	–	0	144	40	212	620
Rus_Mu	0.001	0.001	–	144	41	195	548
Rus_Me	0.006	0.006	0.006	–	113	224	313
Veps	0.006	0.007	0.006	0.006	–	215	388
Komi_Pr	0.011	0.010	0.010	0.009	0.012	–	334
Komi_Izh	0.014	0.014	0.013	0.011	0.014	0.014	–

* Pairwise F_ST_ values are indicated below the diagonal and the number of SNPs is indicated above it. The abbreviations of populations are the same as in Figure 1.

None of the SNPs analyzed showed significant (*P*<3×10^–7^) differences in allele frequencies between populations from the Kursk, Murom, and Tver regions, but 144 to 172 SNPs in each of these populations differed from those of Russians from the Mezen region. The highest number of SNPs with large differences in allele frequencies was found between Izhemski Komi and populations from the Kursk, Murom, and Tver regions (Table 1).

**Figure 3:**
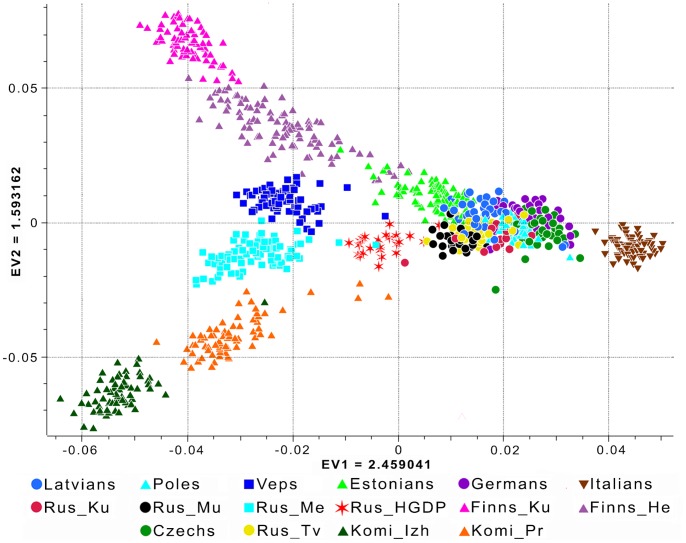
Principal component analysis of the combined autosomal genotypic data of individuals from Russia and seven European countries (Finnland, Estonia, Latvia, Poland, Czech Republic, Germany [5] and Italia [22]). The first two PCs are shown. The color legend for the predefined population labels is indicated within the plot. Population designations are the same as in Figure 1.

To understand the place of Russians, Komi, and Veps on the genetic canvas of Europe, we combined their genotypes with the genotypic data of several European populations (Finns, Estonians, Latvians, Poles, Czechs and Germans, as well as Italians, who are the most distant from our populations [5]). The results of PCA performed on this extended number of samples are shown in Figure 3, and may be described as having an “airplane”-like structure with the two wings represented by the Finnish (upper left), and Komi (lower left) samples. A comparison of the resulting genetic map, with the results presented by Nelis et al. [5], shows that the populations from one of the vertices of the latter are now located at the intersection formed by the two genetic “wings”. Russians from Murom, Kursk, and Tver were also placed at this intersection. However, Russians from Mezen were located outside this intersection. This population, together with the Finnic-speaking Veps, was located in the space between the Finnish and Komi “wings” on the chart. Taking into account the genetic differences found for Mezen Russians among the other Russian populations studied here, a Russian-only sample from the HGDP set was also included in the analysis. The HGDP Russians were also from the Archangelsk region (Kargopol district), but their location is geographically closer to samples of populations from central regions of European Russia (Figure 1). This is reflected in their intermediate position on the PC plot (Figure 3) and lower pairwise F_ST_ values (0.004 against Mezen and 0.002 against the Russians from Kursk, Murom, and Tver regions) (Table S1, Figure S3).

**Figure 4:**
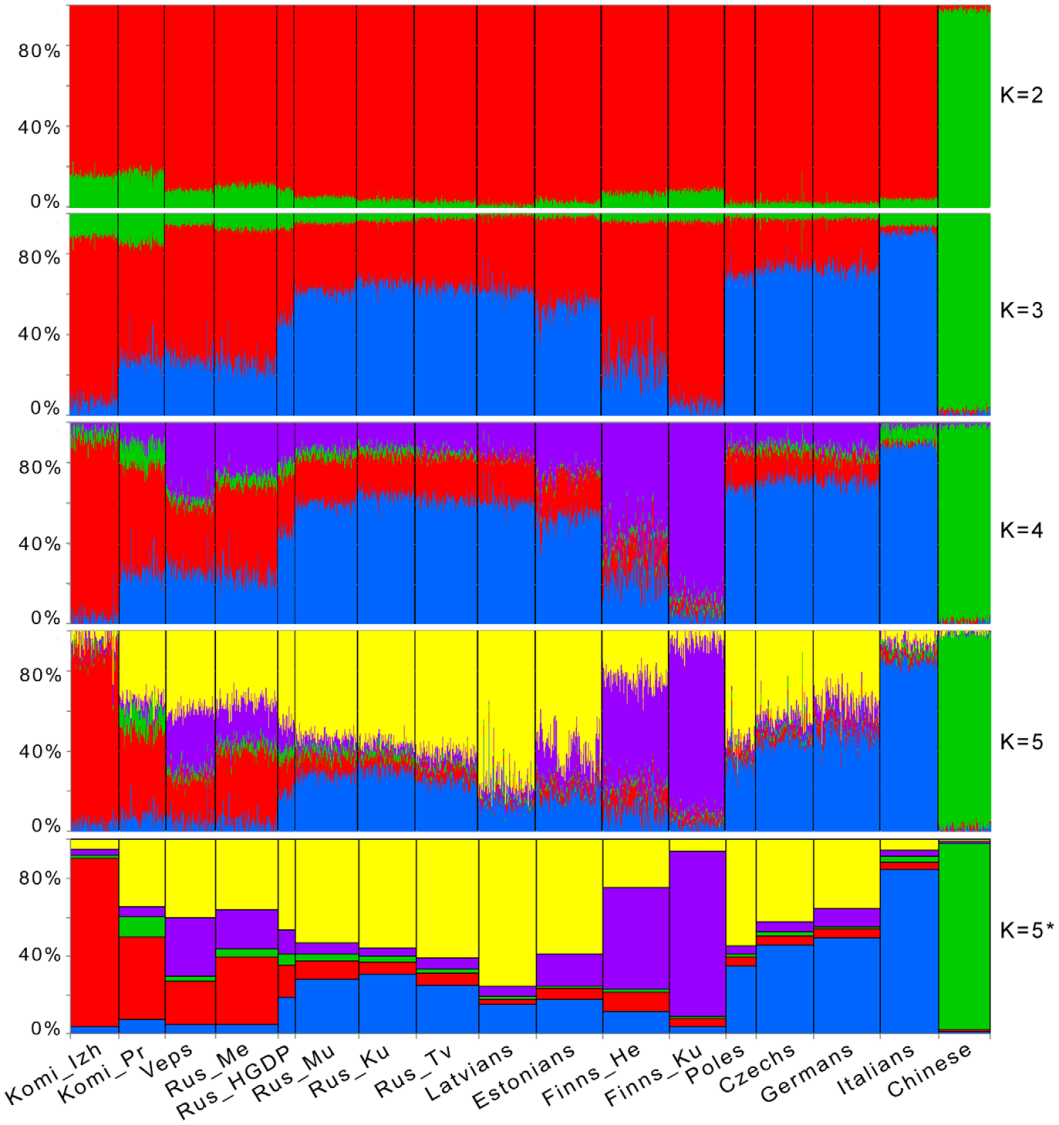
ADMIXTURE clustering of individuals from the populations studied. Results obtained at K = 2 to 5 are shown. Each individual is represented by a vertical line composed of colored segments, in which each segment represents the proportion of an individual’s ancestry derived from one of the K ancestral populations. Individuals are grouped by population (labeled on the bottom of the graph). In addition to populations used in principal component analysis, a Chinese sample (Han Chinese from Beijing [22]) was included. The results at K = 5 are also accompanied by average ancestral proportions by population (*). Population designations are the same as in Figure 1.

To further explore the population structure, a model-based structure-like analysis using the ADMIXTURE software was performed [25]. This analysis considers the genome of each individual as having originated from several hypothetical ancestral populations, the number of which (K) could be specified. In addition to populations already used in PCA, a Chinese sample was included to check for the potential presence of East Asian admixture. We ran ADMIXTURE at K = 2 to 12. At K = 2, only the population groups corresponding to Europe and Asia were separated (Figure 4). Subtle variations detected in this analysis were possibly due to the differences in the proportion of East Asian ancestry, which was present in all European populations included in this study, but had a higher average contribution in Komi samples. Subcontinental patterns appeared at K = 3: one ancestry component was the most abundant in Izhemski Komi and Finns from Kuusamo (red) and a different component (blue) was at the maximum in the Italian population (Figure 4). At K = 4, the red component has diverged into two parts and distinguished Finns (purple) from Komi (red). These results match closely with the population structure revealed by the PCA, where they corresponded to the genetic “wings” described in Figure 3. Mezen Russians and Veps exhibited the highest proportions of both red and purple ancestry components, differing only in their ratios, which were the opposite of each other (henceforth, we will refer to these crucial components as Komi and Finnic). Russians from the HGDP are found at the intermediate position between Mezen and other Russians, with lower proportions of Komi and Finnic components and a higher proportion of the blue component, most common in Italians, compared with the Mezen Russians. At K = 5, a new component is found (yellow), with a high proportion in most of the populations, with the exception of Izhemski Komi, Finns, and Italians. The proportions of Komi and Finnic ancestries were significantly reduced for many central and eastern European populations, but remained high in Veps and Mezen Russians. K = 5 was the observation threshold for subcontinental patterns of genetic variation. At higher K values (K = 6 to 8), we observed the subsequent separations of the populations of Priluzski Komi, Veps, and Mezen Russians (Figure S4). The situation in which a new ancestry component introduced for the next K value differentiated only a single population was considered as being less informative for the hierarchical comparisons of populations [33], [34]. Therefore, although the lowest cross-validation errors were observed at K = 7 (Figure S5), our further discussion will focus on the results of clustering at K = 5.

To explore the potential effect of population demographics on the population structures identified, ROH were compared across populations. ROH may indicate prolonged isolation and a reduced population size [29], [35]. Here, we analyzed ROH longer than 1,500 kb as being the most informative [29]. Using the thresholds for SNP density along a ROH tract (≥ 1 SNP per 50 kb, with a gap size ≤ 100 kb), the total number of ROH in 16 populations (the Chinese sample was not included) was 1,298, with a mean population number of ROH (nROH) of 0.20–2.68 per individual. The population average of the cumulative ROH length (cROH) per individual ranged from 0.43 to 6.31 Mb (Table 2). The use of the alternative definition of ROH, which allows the screening of ROH across various SNPs, resulted in an increase in both the number and length of ROH, which ranged between 6.77 and 17.21 Mb for nROH and 19.62 and 71.83 Mb for cROH. Regardless of the variations in the analysis, the highest nROH and cROH values were found in Izhemski Komi and in the Finnish sample from Kuusamo. Intermediate estimates were observed in Priluzski Komi, Veps, Finns from Helsinki, and Mezen Russians. Other populations had lower nROH and cROH values. An analysis of LD decay across genomes showed that Izhemski Komi and Finns from Kuusamo also exhibited elevated LD (Figure S6). Concomitantly, Priluzski Komi, Veps, Mezen Russians, and Finns from Helsinki exhibited only slightly elevated LD and were more comparable to the level observed in other European samples, including the remaining Russian samples.

**Table 2:** Summary of ROH statistics of 16 European populations.

Population	nROH	cROH	nROH*	cROH*
Italians	0.19	0.4	6.27	19.64
Germans	0.2	0.43	6.77	19.62
Rus_Ku	0.28	0.65	7.88	24.4
Czechs	0.35	0.64	7.23	19.79
Rus_Mu	0.39	0.98	7.97	27.41
Rus_HGDP	0.44	0.83	8.92	25.15
Rus_Tv	0.49	1.12	9.34	28.05
Poles	0.51	1.11	8.51	27.43
Latvians	0.58	1.08	10.62	29.56
Estonians	0.61	1.45	9.95	33.26
Finns_He	1.13	2.32	12.85	41.47
Rus_Me	1.63	3.81	13.02	51.42
Veps	1.72	3.87	14.77	54.29
Komi_Ob	1.77	3.94	13.13	52.17
Finns_Ku	2.24	4.95	16.58	58.76
Komi_Izh	2.68	6.31	17.21	71.83

* ROH calculated without the thresholds for SNP density and length of the gaps along a ROH tract. The abbreviations of populations are the same as in Figure 1.

## Discussion

In this study, we used genome-wide SNP data to analyze the population genetic structure of Russians, Veps, and Komi. The samples under investigation covered territories in the northeastern Europe, not been included in previous analyses.

The results obtained revealed no substantial genetic stratification within Russians from central–southern regions of European Russia (i.e., samples from the Kursk, Murom, and Tver regions). These three populations were clustered in close proximity to other populations from central–eastern Europe. In contrast, a sample from the northern Archangelsk region of Russia, Mezen Russians, was clearly distant from those of Kursk, Murom, and Tver. These data are in good agreement with earlier data obtained for polymorphisms of the Y-chromosome [14], [15], [36], [37] and several autosomal loci [16], [38], [39]. It has been proposed that the genetic specificity of northern Russians is because of admixture with Finno-Ugric populations. The results of our ADMIXTURE analysis suggest that, although they descended historically from the Novgorod Russians, Mezen Russians admixed significantly with both Finnic-speaking and Komi populations (Komi belongs to the different linguistic branch of the Finno-Ugric family, the Permian branch). The estimated proportion of Komi ancestry in Mezen Russians was higher than the Finnic proportion. This might be explained by either a more extensive or a later admixture with Komi people. The existing anthropological data favor the latter explanation, proposing a two-staged inclusion of Finno-Ugric elements during the ethnogenesis of Northern Russians, in which Komi elements were included last [40]. Both the Komi and Finnic ancestry components occurred at lower proportions in other Russians, as well as in the populations of Poles, Czechs, Germans, and Italians, which are geographically distant from Finns and Komi. The proportions of Komi and Finnic components were also low in Latvians, but not in Estonians, among whom the proportion of Finnic ancestry was relatively high.

The Veps were another population that exhibited an increased percentage of both Komi and Finnic ancestries. The high level of Finnic ancestry is evidently characteristic of this population, as they belong to the same linguistic community, the Finnic-speaking community, as Finns do. The higher level of Komi ancestry in this population compared with that of Finns and Estonians could be from admixture of Veps (Ves) with Komi (ancient Permians) in the 11^th^–14^th^ centuries, when Komi lived westward of their current territory and were the neighbors of Veps [41].

As for the Komi themselves, it has been proposed [42] that their ethnogenesis was influenced by Finnic (e.g., Veps or “Chud”) and Russian people. The evaluation of the impact of Finnic people in the context of Finnic ancestry revealed that the corresponding component was not represented at a high proportion in the Komi samples studied. The impact of Russians on the ethnogenesis of Komi seems to be indicated by the yellow component. It was abundant in Priluzski Komi (Figure 4), which is in good agreement with the population history of this region – the basin of the Luza river, where Russian people resided as far back as the 13^th^–14^th^ centuries [41]. In contrast to the Priluzski Komi, Komi component was overrepresented in the ancestry of the Izhemski Komi, accounting for more than 80% of the total ancestry (86% at K = 5). Historical records show that the first mention of the current center of Izhemski Komi, the Izhma village, occurred at the end of the 16^th^ century and that Izhma was founded mainly by a group of Vimski Komi. Later, some Russian and Nenets families joined them [41], [43]. Nenets were not studied here. Although the ADMIXTURE components depend on the samples included, a minimal influence of the genetics of Nenets on the results of clustering can be proposed. Here, we can refer to both the existing data on the absence of (or very limited) genetic relationships between the Nenets and the populations listed (including Komi) [15], [44], [45], and the results of our analyses, which indicate the genetic isolation of the Izhemski Komi. Evidence of the latter stemmed both from pairwise F_ST_ values, which were the same between Izhemski Komi and both Priluzski Komi, who shared the same ethnic territory, and the geographically distant Finns from Helsinki, and from their higher parameters of ROH estimated.

Both nROH and cROH have been shown to be higher in northern Europeans compared to their southern counterparts, which is consistent with the smaller effective population size and lower population density in northern Europe [35]. In our study, all northern samples (Mezen Russians, Veps, and both Komi samples) were also characterized by higher nROH and cROH compared to Russians from the central part of the Russian Plain and most of the European populations tested. However, the Izhemski Komi had the highest nROH and cROH, comparable to the values calculated in the sample from Kuusamo, the known Finnish isolate [46]. Similar to the Finns from Kuusamo, the Izhemski Komi exhibited elevated LD. Taking into account the history of the Komi people, the recorded genetic distinction of the Izhemski Komi can be due to the increased stability of their community life reinforced by the advanced type of traditional economy, including reindeer breeding [47]. Reindeer breeding was adopted by this group from the Nenets and currently differentiates the Izhemski Komi from the other Komi groups.

In summary, we reported results of the first genome-wide autosomal SNP-based study of the population structure of European Russia, in which samples of Russians, Veps, and Komi were analyzed. The data obtained strongly supports the results of earlier genetic studies, based either on Y-chromosome polymorphisms or on a limited number of autosomal markers, and suggested a genetic distinction of the northern Russian populations. Here, we were able to show clearly that this distinction was attributed to admixture with Finno-Ugric populations. The second important finding of our work was the context of that admixture. Our data on Komi population structure led us to consider this group as the second pole of genetic diversity in northern Europe (in addition to the pole occupied by Finns). Although we understand that the picture of the genetic structure of populations from European Russia obtained is still sparse, we propose that populations (ethnic groups) located between those two poles will have different proportions of Komi and Finnic ancestries (e.g., Veps and Mezen Russians).

## Supporting Information

Figure S1 **Scree plots for eigenvalues of components 1 to 25 from the principal component analysis: (A)** individuals from Russia, (B) individuals from Russia and selected samples from seven European countries.
(TIF)

Figure S2 **Principal component analysis of the autosomal genotypic data of individuals from European Russia.** PC3 and PC4 are shown. Population designations are the same as in Figure 1.
(TIF)

Figure S3 **Multidimensional scaling analysis (two dimensions) of pairwise F_ST_ among 16 European populations.** The F_ST_ matrix from Table S1 was used as an input for the analysis.
(TIF)

Figure S4 **Results of ADMIXTURE clustering at K = 6 to 8.** The number of populations and their order are the same as at Figure 4.
(TIF)

Figure S5 **Cross-validation plot for 16 populations from the ADMIXTURE analysis.** The plot displays the cross-validation error versus K. The results of eight runs with different random seeds are presented.
(TIF)

Figure S6 **The decay of LD across the genomes of the populations from Russia and the European reference samples.** The samples of Poles and Russians from the HGDP were not included because of their smaller sample size. The Italian sample was also excluded (its merging with other samples resulted in a significant decrease in the number of SNPs).
(TIF)

Table S1 **F_ST_ statistics calculated in pairs of all European populations analyzed.**
(DOC)

## References

[1] Seldin MF, Shigeta R, Villoslada P, Selmi C, Tuomilehto J, et al (2006) Gregersen PK. European population substructure: clustering of northern and southern populations. PLoS Genet 2: e143. 17044734 10.1371/journal.pgen.0020143 PMC1564423

[2] Salmela E, Lappalainen T, Fransson I, Andersen PM, Dahlman-Wright K, et al (2008) Genome-wide analysis of single nucleotide polymorphisms uncovers population structure in Northern Europe. PLoS One 3: e3519. 18949038 10.1371/journal.pone.0003519 PMC2567036

[3] Li JZ, Absher DM, Tang H, Southwick AM, Casto AM, et al (2008) Worldwide Human Relationships Inferred from Genome-Wide Patterns of Variation. Science 319: 1100–4. 18292342 10.1126/science.1153717

[4] Novembre J, Johnson T, Bryc K, Kutalik Z, Boyko AR, et al (2008) Genes mirror geography within Europe. Nature 456: 98–101. 18758442 10.1038/nature07331 PMC2735096

[5] Nelis M, Esko T, Mägi R, Zimprich F, Zimprich A, et al (2009) Genetic Structure of Europeans: A View from the North–East. PLoS One 4: e5472. 19424496 10.1371/journal.pone.0005472 PMC2675054

[6] Tian C, Plenge RM, Ransom M, Lee A, Villoslada P, et al (2008) Analysis and application of European genetic substructure using 300 K SNP information. PLoS Genet 4: e4. 18208329 10.1371/journal.pgen.0040004 PMC2211544

[7] Price AL, Helgason A, Palsson S, Stefansson H, St Clair D, et al (2009) PLoS Genet. 5: e1000505. 10.1371/journal.pgen.1000505 PMC2684636 19503599

[8] Humphreys K, Grankvist A, Leu M, Hall P, Liu J, et al (2011) The Genetic Structure of the Swedish Population. PLoS One 6: e22547. 21829632 10.1371/journal.pone.0022547 PMC3150368

[9] O'Dushlaine CT, Morris D, Moskvina V, Kirov G, Consortium IS, et al (2010) Population structure and genome-wide patterns of variation in Ireland and Britain. Eur J Hum Genet 18: 1248–54. 20571510 10.1038/ejhg.2010.87 PMC2987482

[10] Pistis G, Piras I, Pirastu N, Persico I, Sassu A, et al (2009) High Differentiation among Eight Villages in a Secluded Area of Sardinia Revealed by Genome-Wide High Density SNPs Analysis. PLoS One 4: e4654. 19247500 10.1371/journal.pone.0004654 PMC2646134

[11] Huyghe JR, Fransen E, Hannula S, Van Laer L, Van Eyken E, et al (2011) A genome-wide analysis of population structure in the Finnish Saami with implications for genetic association studies. Eur J Hum Genet 19: 347–52. 21150888 10.1038/ejhg.2010.179 PMC3062008

[12] Lao O, Lu TT, Nothnagel M, Junge O, Freitag-Wolf S, et al (2008) Correlation between genetic and geographic structure in Europe. Curr Biol 18: 1241–1248. 18691889 10.1016/j.cub.2008.07.049

[13] Russian Census. Available: http://www.gks.ru/free_doc/new_site/perepis2010/croc/perepis_itogi1612.htm. Accessed 2012 Dec 21.

[14] Khrunin AV, Bebiakova NA, Ivanov VP, Solodilova MA, Limborskaia SA (2005) Polymorphism of Y-chromosomal microsatellites in Russian populations from the northern and southern Russia as exemplified by the populations of Kursk and Arkhangel'sk Oblast. Genetika 41: 1125–31. 16161634

[15] Balanovsky O, Rootsi S, Pshenichnov A, Kivisild T, Churnosov M, et al (2008) Two sources of the Russian patrilineal heritage in their Eurasian context. Am J Hum Genet 82: 236–250. 18179905 10.1016/j.ajhg.2007.09.019 PMC2253976

[16] Flegontova OV, Khrunin AV, Lylova OI, Tarskaia LA, Spitsyn VA, et al (2009) Haplotype frequencies at the DRD2 locus in populations of the East European Plain. BMC Genet 10: 62. 19793394 10.1186/1471-2156-10-62 PMC2765450

[17] Alexeeva TI (Editor) (2002) Eastern Slavs. Anthropology and ethnic history. Moscow: Nauchny mir. 342 p.

[18] Pimenov VV (1965) Veps: A study of ethnic history and genesis of culture. Moscow-Leningrad: Nauka. 264 p.

[19] Savel’eva EA (Editor) (2001) Atlas of the Komi Republic. Moscow: Dizain. Moscow: Inter’er Kartographiya. 552 p.

[20] Khrunin A, Verbenko D, Nikitina K, Limborska S (2007) Regional differences in the genetic variability of Finno-Ugric speaking Komi populations. Am J Hum Biol 19: 741–50. 17691096 10.1002/ajhb.20620

[21] Milligan BG (1998) Total DNA isolation. In: Hoelzel AR, editor. Molecular Genetic Analysis of Populations. Oxford: Oxford University Press. pp. 29–60.

[22] The HapMap 3 genotype data (R2 B356 FWD). Available for users of SNP and Variation Suite software package (Golden Helix, Bozeman, MT, USA). Accessed 2012 April 5.

[23] The HGDP-CEPH diversity panel. Available: http://www.cephb.fr/en/hgdp/. Accessed 2012 April 10.

[24] Patterson N, Price AL, Reich D (2006) Population Structure and Eigenanalysis. PLoS Genet 2: e190. 17194218 10.1371/journal.pgen.0020190 PMC1713260

[25] Alexander DH, Novembre J, Lange K (2009) Fast model-based estimation of ancestry in unrelated individuals. Genome Res 19 1655–64. 19648217 10.1101/gr.094052.109 PMC2752134

[26] Biswas S, Scheinfeldt LB, Akey JM (2009) Genome-wide insights into the patterns and determinants of fine-scale population structure in humans. Am J Hum Genet 84: 641–50. 19442770 10.1016/j.ajhg.2009.04.015 PMC2681007

[27] Luca D, Ringquist S, Klei L, Lee AB, Gieger C, et al (2008) On the use of general control samples for genome-wide association studies: genetic matching highlights causal variants. Am J Hum Genet 82: 453–63. 18252225 10.1016/j.ajhg.2007.11.003 PMC2427172

[28] Alexander DH, Lange K (2011) Enhancements to the ADMIXTURE algorithm for individual ancestry estimation. BMC Bioinformatics 12: 246. 21682921 10.1186/1471-2105-12-246 PMC3146885

[29] McQuillan R, Leutenegger AL, Abdel-Rahman R, Franklin CS, Pericic M, et al. (2008) Runs of homozygosity in European populations. Am J Hum Genet 83 359–372. 10.1016/j.ajhg.2008.08.007 PMC2556426 18760389

[30] Spain SL, Cazier JB, CORGI Consortium, Houlston R, Carvajal-Carmona L, et al (2009) Colorectal cancer risk is not associated with increased levels of homozygosity in a population from the United Kingdom. Cancer Res 69: 7422–9. 19723657 10.1158/0008-5472.CAN-09-0659

[31] Howrigan DP, Simonson MA, Keller MC (2011) Detecting autozygosity through runs of homozygosity: a comparison of three autozygosity detection algorithms. BMC Genomics 12: 460. 21943305 10.1186/1471-2164-12-460 PMC3188534

[32] Purcell S, Neale B, Todd-Brown K, et al (2007) PLINK: a toolset for whole-genome association and population-based linkage analysis. Am J Human Genet 81: 559–75. 17701901 10.1086/519795 PMC1950838

[33] Behar DM, Yunusbayev B, Metspalu M, Metspalu E, Rosset S, et al (2010) The genome-wide structure of the Jewish people. Nature 466: 238–242. 20531471 10.1038/nature09103

[34] Rasmussen M, Li Y, Lindgreen S, Pedersen JS, Albrechtsen A, et al (2010) Ancient human genome sequence of an extinct Palaeo-Eskimo. Nature 463: 757–62. 20148029 10.1038/nature08835 PMC3951495

[35] Nothnagel M, Lu TT, Kayser M, Krawczak M (2010) Genomic and geographic distribution of SNP defined runs of homozygosity in Europeans. Hum Mol Genet 19: 2927–35. 20462934 10.1093/hmg/ddq198

[36] Malyarchuk B, Derenko M, Grzybowski T, Lunkina A, Czarny J, et al (2004) Differentiation of Mitochondrial DNA and Y chromosomes in Russian Populations. Hum Biol 76: 877–900. 15974299 10.1353/hub.2005.0021

[37] Mirabal S, Regueiro M, Cadenas AM, Cavalli-Sforza LL, Underhill PA, et al (2009) Y-chromosome distribution within the geo-linguistic landscape of northwestern Russia. Eur J Hum Genet 17: 1260–73. 19259129 10.1038/ejhg.2009.6 PMC2986641

[38] Khrunin AV, Khokhrin DV, Limborskaia SA (2008) Glutathione-S-transferase gene polymorphism in Russian populations of European Russia Genetika 47, 1565–8. 19062541

[39] Verbenko DA, Slominsky PA, Spitsyn VA, Bebyakova NA, Khusnutdinova EK, et al (2006) Polymorphisms at locus D1S80 and other hypervariable regions in the analysis of Eastern European ethnic group relationships. Ann Hum Biol 33: 570–84. 17381055 10.1080/03014460601012077

[40] Alexeev VP (1969) The origin of nations of Eastern Europe. Moscow: Nauka. 324 p.

[41] Zherebtsov LN (1982) Historical and cultural relationships of Komi with their neighbors. Moscow: Nauka. 224 p.

[42] Savel’eva EA (Editor) (1997) Archeology of Komi Republic. Moscow: DIK. 758 p.

[43] Zherebtsov IL (1996) The population of Komi territory in the second half of XVI century to the beginning of XVIII century. Yekaterinburg: UrO RAN. 258 p.

[44] Cavalli-Sforza L, Menozzi P, Piazza A (1996) The history and geography of human genes. Princeton: Princeton University Press. 414 p.

[45] Karafet TM, Osipova LP, Gubina MA, Posukh OL, Zegura SL, et al (2002) High levels of Y-chromosome differentiation among native Siberian populations and the genetic signature of a boreal hunter-gatherer way of life. Hum Biol 74: 761–89. 12617488 10.1353/hub.2003.0006

[46] Varilo T, Laan M, Hovatta I, Wiebe V, Terwilliger JD, et al (2000) Linkage disequilibrium in isolated populations: Finland and a young sub-population of Kuusamo. Eur J Hum Genet 8: 604–12. 10951523 10.1038/sj.ejhg.5200482

[47] Konakov ND, Kotov OV (1991) Ethnoarealic Komi group: the formation and current ethnic and cultural status. Moscow: Nauka. 232 p.

