# Supplementary figures and images for: A Genome-Wide Analysis of Populations from European Russia Reveals a New Pole of Genetic Diversity in Northern Europe
Source: PLoS One. 2013 Mar 7;8(3):e58552. doi: 10.1371/journal.pone.0058552 (PMC3591355; doi:10.1371/journal.pone.0058552)

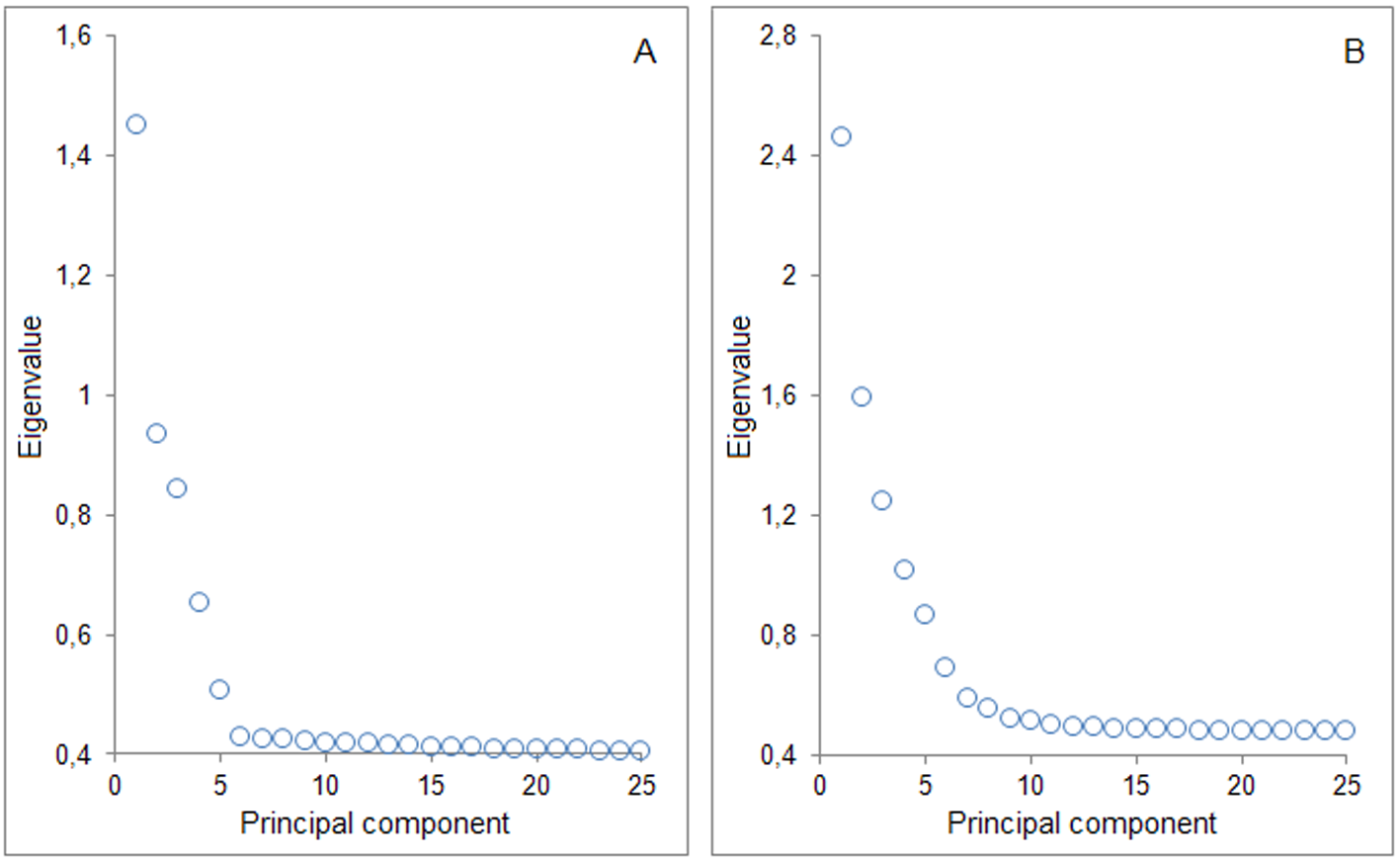

Supplement: Figure S1 — Scree plots for eigenvalues of components 1 to 25 from the principal component analysis: (A) individuals from Russia, (B) individuals from Russia and selected samples from seven European countries. (TIF) [file pone.0058552.s001.tif]

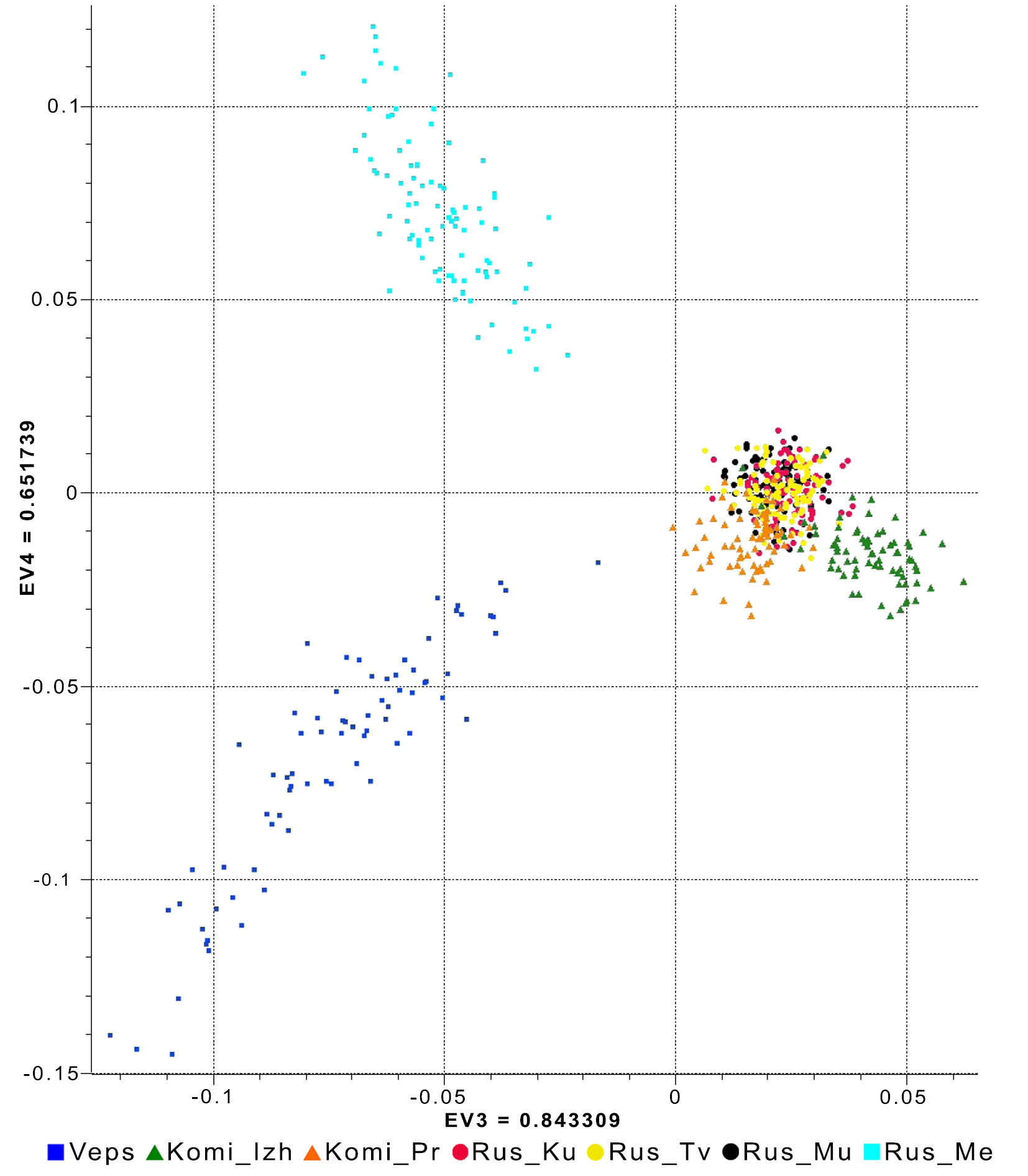

Supplement: Figure S2 — Principal component analysis of the autosomal genotypic data of individuals from European Russia. PC3 and PC4 are shown. Population designations are the same as in Figure 1. (TIF) [file pone.0058552.s002.tif]

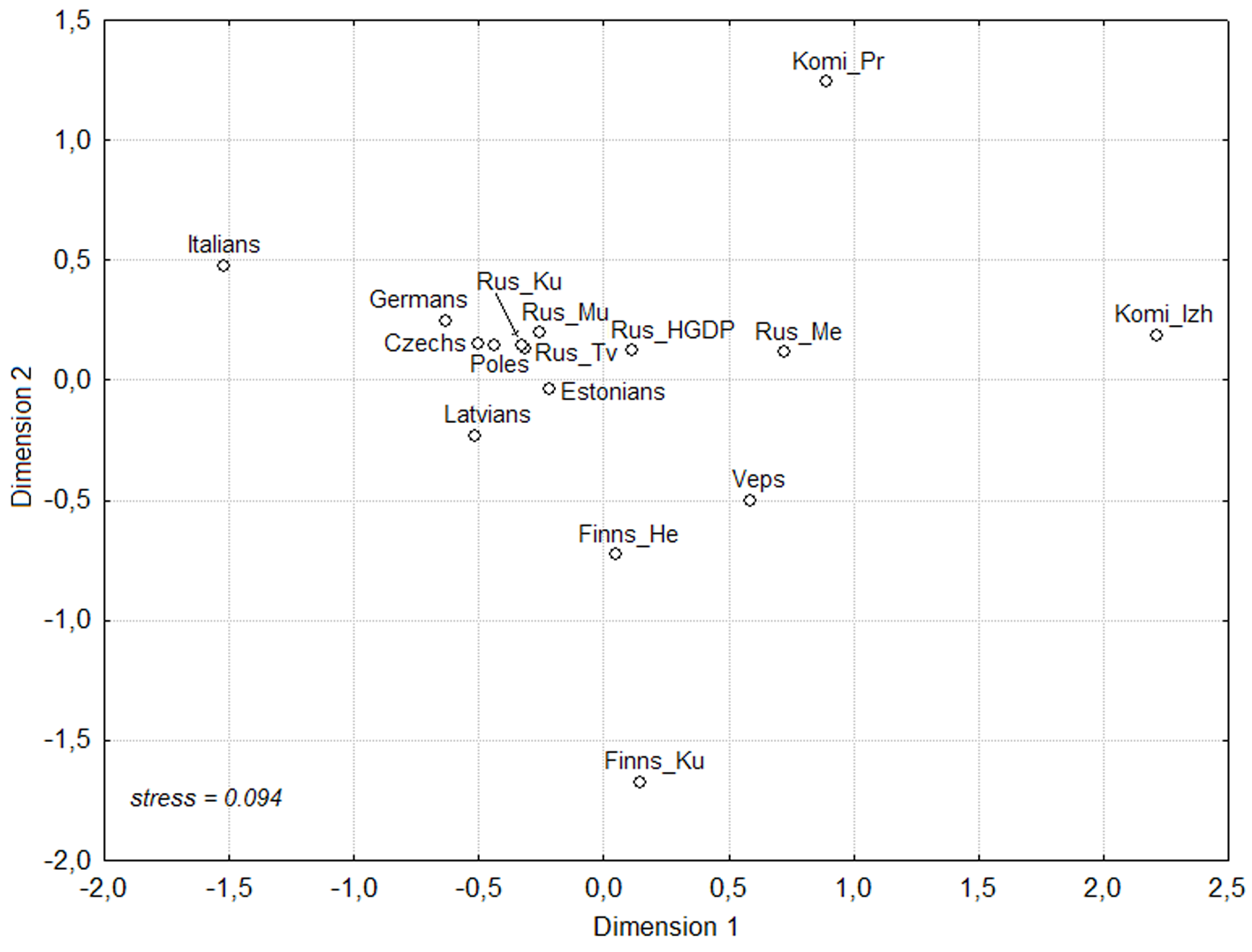

Supplement: Figure S3 — Multidimensional scaling analysis (two dimensions) of pairwise FST among 16 European populations. The FST matrix from Table S1 was used as an input for the analysis. (TIF) [file pone.0058552.s003.tif]

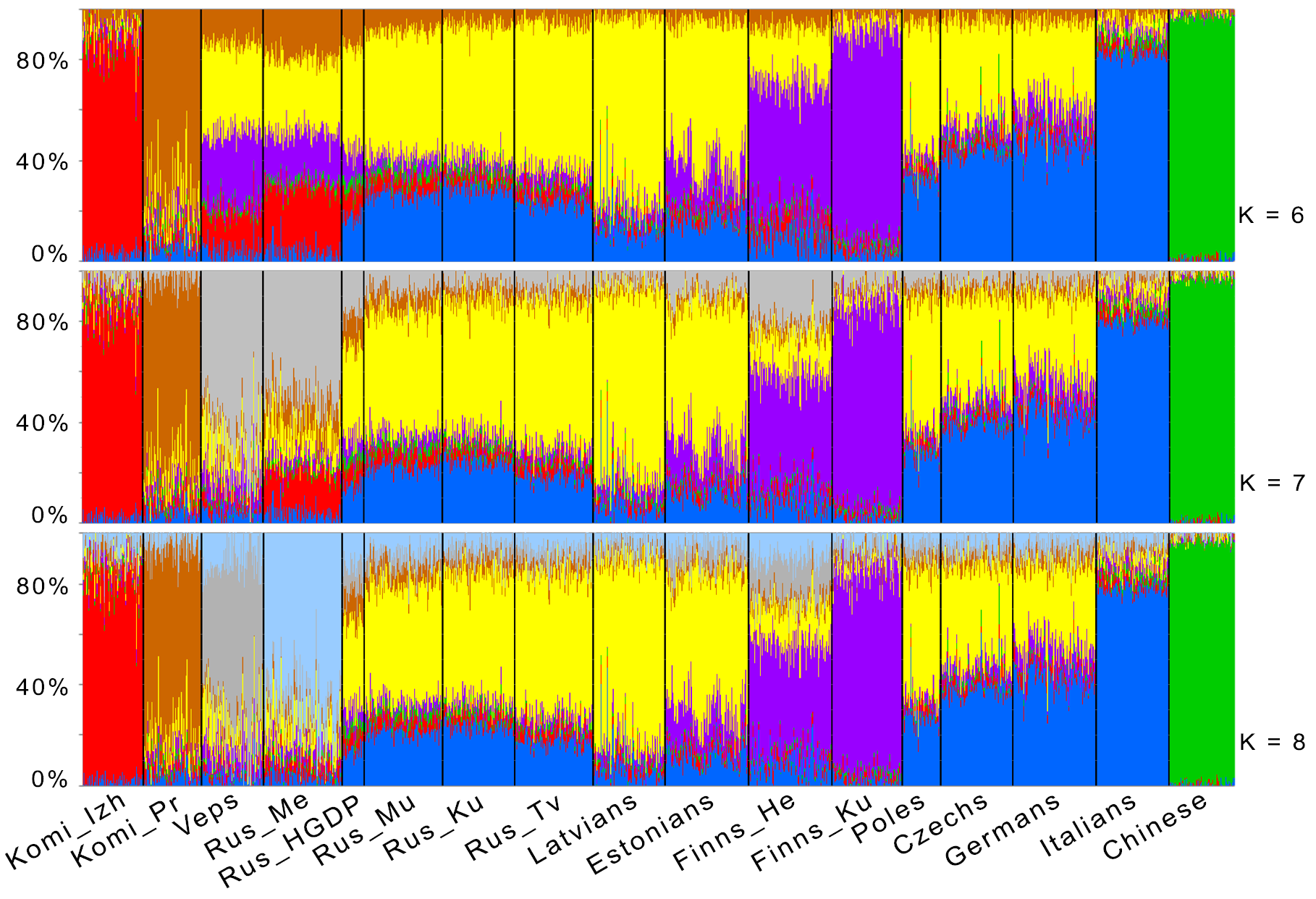

Supplement: Figure S4 — Results of ADMIXTURE clustering at K = 6 to 8. The number of populations and their order are the same as at Figure 4. (TIF) [file pone.0058552.s004.tif]

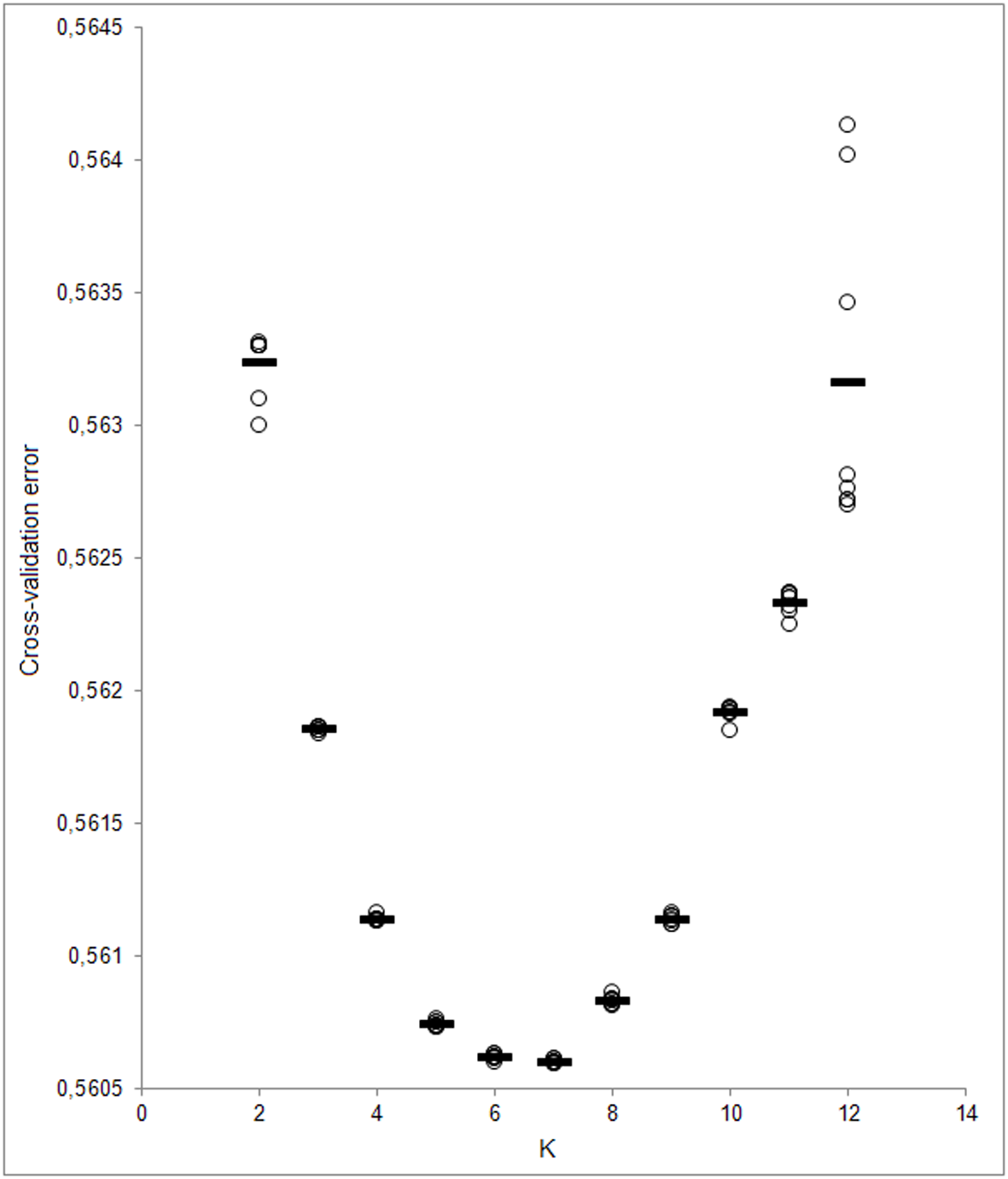

Supplement: Figure S5 — Cross-validation plot for 16 populations from the ADMIXTURE analysis. The plot displays the cross-validation error versus K. The results of eight runs with different random seeds are presented. (TIF) [file pone.0058552.s005.tif]

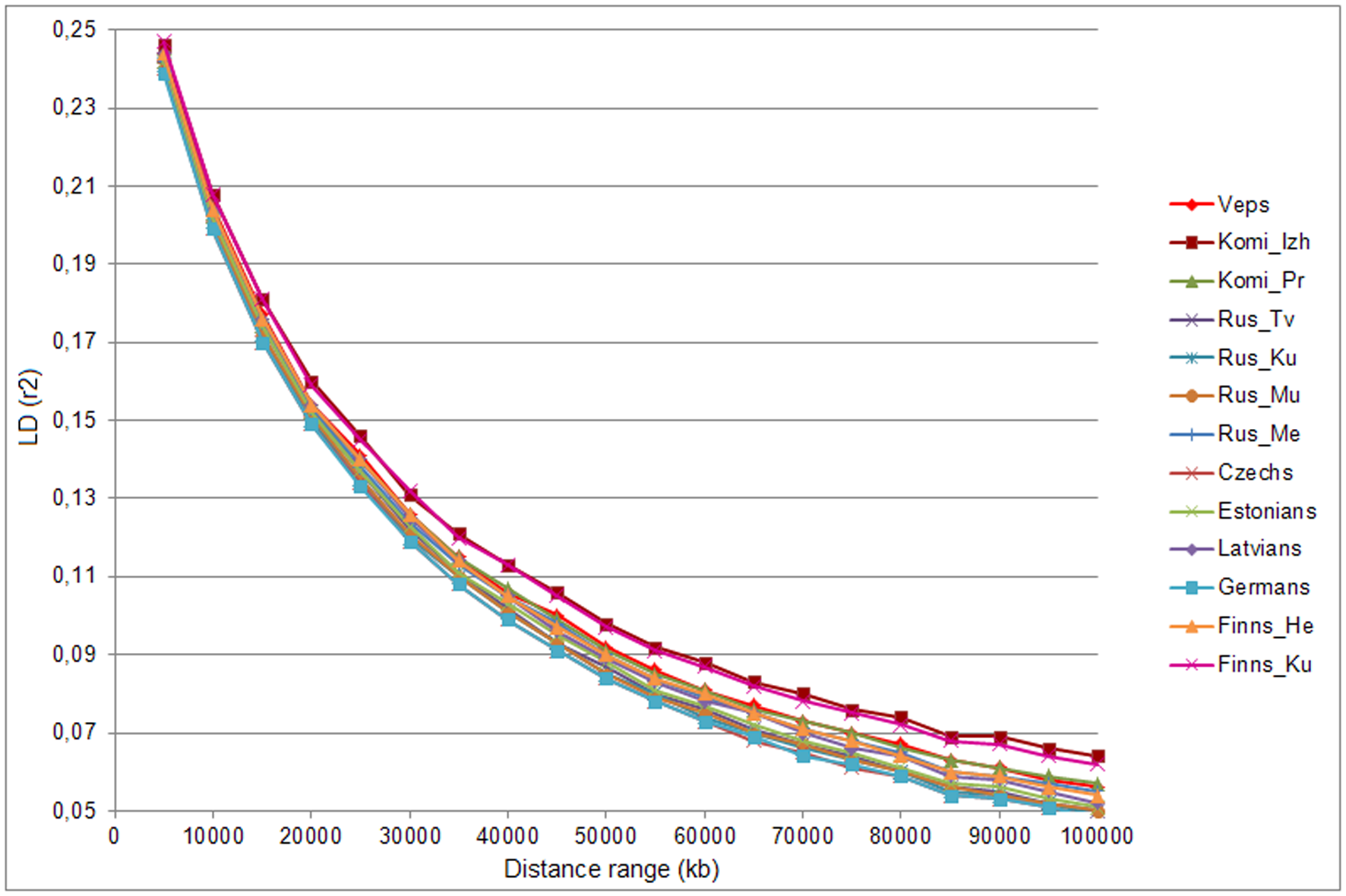

Supplement: Figure S6 — The decay of LD across the genomes of the populations from Russia and the European reference samples. The samples of Poles and Russians from the HGDP were not included because of their smaller sample size. The Italian sample was also excluded (its merging with other samples resulted in a significant decrease in the number of SNPs). (TIF) [file pone.0058552.s006.tif]
