# Supplementary material for: A Genome-Wide Analysis of Populations from European Russia Reveals a New Pole of Genetic Diversity in Northern Europe
Source: PLoS One. 2013 Mar 7;8(3):e58552. doi: 10.1371/journal.pone.0058552 (PMC3591355; doi:10.1371/journal.pone.0058552)
Supplement: Table S1 — FST statistics calculated in pairs of all European populations analyzed. (DOC) [file pone.0058552.s007.doc]

**Table S1. F_ST_ statistics calculated in pairs of all European populations analyzed***

|  | Veps | Komi_Izh | Komi_Pr | Rus_Tv | Rus_Ku | Rus_Mu | Rus_Me | Czechs | Estonians | Latvians | Germans | Finns_He | Finns_Ku | Poles | Rus_HGDP | Italians |
| --- | --- | --- | --- | --- | --- | --- | --- | --- | --- | --- | --- | --- | --- | --- | --- | --- |
| Veps | — |  |  |  |  |  |  |  |  |  |  |  |  |  |  |  |
| Komi_Izh | 0.014 | — |  |  |  |  |  |  |  |  |  |  |  |  |  |  |
| Komi_Pr | 0.012 | 0.014 | — |  |  |  |  |  |  |  |  |  |  |  |  |  |
| Rus_Tv | 0.007 | 0.015 | 0.011 | — |  |  |  |  |  |  |  |  |  |  |  |  |
| Rus_Ku | 0.007 | 0.015 | 0.011 | 0.000 | — |  |  |  |  |  |  |  |  |  |  |  |
| Rus_Mu | 0.006 | 0.014 | 0.010 | 0.001 | 0.001 | — |  |  |  |  |  |  |  |  |  |  |
| Rus_Me | 0.006 | 0.010 | 0.009 | 0.006 | 0.006 | 0.006 | — |  |  |  |  |  |  |  |  |  |
| Czechs | 0.008 | 0.016 | 0.012 | 0.001 | 0.001 | 0.001 | 0.007 | — |  |  |  |  |  |  |  |  |
| Estonians | 0.006 | 0.014 | 0.011 | 0.001 | 0.001 | 0.002 | 0.006 | 0.002 | — |  |  |  |  |  |  |  |
| Latvians | 0.008 | 0.017 | 0.013 | 0.002 | 0.002 | 0.003 | 0.008 | 0.003 | 0.001 | — |  |  |  |  |  |  |
| Germans | 0.008 | 0.016 | 0.012 | 0.002 | 0.002 | 0.002 | 0.008 | 0.001 | 0.003 | 0.004 | — |  |  |  |  |  |
| Finns_He | 0.006 | 0.014 | 0.011 | 0.006 | 0.006 | 0.005 | 0.006 | 0.006 | 0.004 | 0.007 | 0.006 | — |  |  |  |  |
| Finns_Ku | 0.011 | 0.019 | 0.016 | 0.012 | 0.012 | 0.012 | 0.011 | 0.013 | 0.010 | 0.013 | 0.012 | 0.005 | — |  |  |  |
| Poles | 0.007 | 0.015 | 0.011 | 0.001 | 0.000 | 0.001 | 0.007 | 0.000 | 0.001 | 0.002 | 0.001 | 0.006 | 0.012 | — |  |  |
| Rus_HGDP | 0.005 | 0.011 | 0.007 | 0.002 | 0.002 | 0.002 | 0.004 | 0.003 | 0.002 | 0.003 | 0.003 | 0.004 | 0.010 | 0.002 | — |  |
| Italians | 0.014 | 0.022 | 0.018 | 0.008 | 0.007 | 0.007 | 0.014 | 0.004 | 0.009 | 0.011 | 0.004 | 0.013 | 0.019 | 0.006 | 0.009 | — |

*The abbreviations of populations are the same as in Figure 1.
